# Is There a Future for Minimal Access and Robots in Cardiac Surgery?

**DOI:** 10.3390/jcdd10090380

**Published:** 2023-09-04

**Authors:** Gloria Faerber, Murat Mukharyamov, Torsten Doenst

**Affiliations:** Department of Cardiothoracic Surgery, Jena University Hospital, Friedrich Schiller University, Am Klinikum 1, 07747 Jena, Germany

**Keywords:** cardiac surgery, minimal access, robotic surgery

## Abstract

Minimally invasive techniques in cardiac surgery have found increasing use in recent years. Both patients and physicians often associate smaller incisions with improved outcomes (i.e., less risk, shorter hospital stay, and a faster recovery). Videoscopic and robotic assistance has been introduced, but their routine use requires specialized training and is associated with potentially longer operating times and higher costs. Randomized evidence is scarce and transcatheter treatment alternatives are increasing rapidly. As a result, the concept of minimally invasive cardiac surgery may be viewed with skepticism. In this review, we examine the current status and potential future perspectives of minimally invasive and robotic cardiac surgery.

## 1. Introduction

It has been more than 70 years since cardiac surgery evolved from a “risky endeavor” with limited chance of success to a standardized procedure used to predictably treat patients with heart disease. The introduction of cardiopulmonary bypass and the implementation of myocardial protection techniques have led to remarkable progress in the field, practically providing a full spectrum of surgical treatment options for all main cardiac pathologies [1]. Over time, techniques have been refined. Surgeons and patients alike began to prioritize reducing surgical trauma and improving cosmetic results. This focus led to the development of less invasive procedures in the cardiac surgical field for valve repair [2,3] and coronary artery bypass graft surgery [4]. During this process, robotic technologies were developed, primarily as a telemanipulator and with improved instrument maneuverability [5]. In more recent years, transcatheter technologies emerged as even less invasive alternatives, which now enable the interventional treatment of coronary and/or valve pathologies as an isolated or combined procedure [6]. As a result, the current times are characterized by a plethora of methods and technologies that are available to treat cardiac diseases. This is a new situation, specifically for cardiac surgery, for which the initial developments had practically no reasonable alternative [1]. Thus, efforts to achieve the same surgical result with a smaller incision or the use of a telemanipulator may be questioned, if there is a fully interventional alternative. In this context, we here review the available evidence for minimally invasive and robotically assisted surgery. We will demonstrate that minimally invasive surgical treatment options are far from becoming obsolete, but are valuable tools for the multidisciplinary heart team and their task to optimize individual patient treatment.

## 2. Definition of Minimally Invasive Cardiac Surgery

There is no universal definition for the term minimally invasive cardiac surgery. In general, normal invasiveness is characterized by the use of sternotomy as an access route to the heart and the use of cardiopulmonary bypass. Thus, off-pump procedures have often been termed minimally invasive cardiac surgery, although sternotomy is still the main access route [7]. However, in the context of this article, minimally invasive cardiac surgery refers to operations with smaller incisions and access to the heart either through partial sternotomy or through mini-thoracotomies.

**Figure 1:**
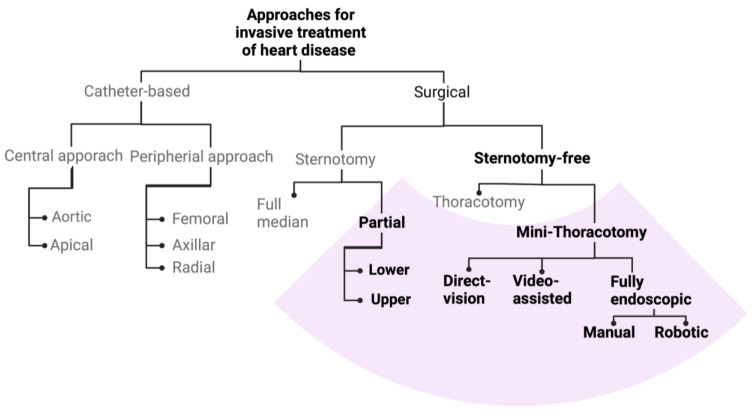
Graphic categorization of the different approaches used for the invasive treatment of heart disease.

**Figure 2:**
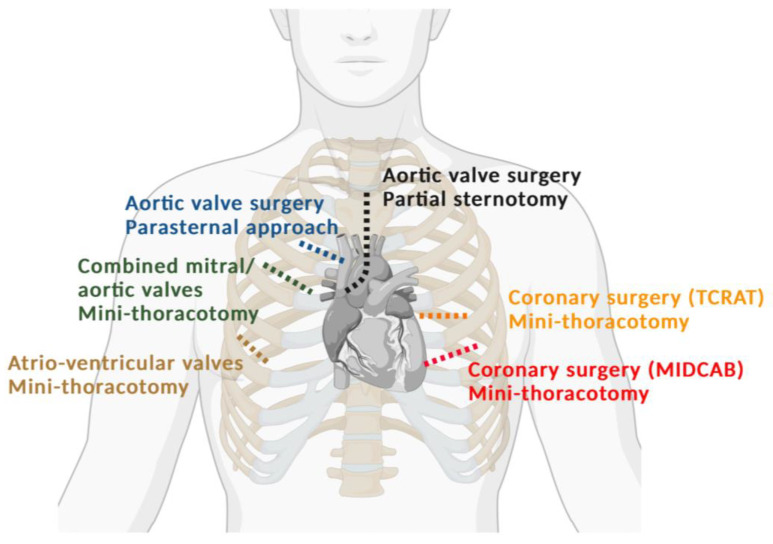
Schematic illustration of minimally invasive access routes to the heart in relation to the intended cardiac surgical procedure.

Figure 1 shows a graphic categorization of the different invasive approaches used to treat heart disease. The marked area encompasses the procedures that are generally summarized as minimally invasive cardiac surgery. These minimally invasive approaches can be sub-classified in partial sternotomy or sternotomy-free approaches. The use of videoscopic or robotic technology is more common in sternotomy-free approaches. Figure 2 schematically illustrates the minimally invasive access routes to the heart in relation to the respective cardiac surgical procedure. For the mitral valve, a mini-thoracotomy has become routine in many centers (60% of all isolated mitral cases were accessed through mini-thoracotomies in Germany in 2022). For the aortic valve, access through partial sternotomy is becoming increasingly popular, with up to 40% of cases in Germany performed through this route [7]. In addition, some centers use parasternal, mini-thoracotomy approaches as their primary access route to the aortic valve [8] Again, modified approaches allow us to address combined pathologies involving not only the mitral and tricuspid valves but also simultaneously correcting the aortic valve [9]. Moreover, in recent years, reports have emerged that describe the combination of two mini-thoracotomies for simultaneous interventions on valves and coronaries, such as bypass grafting of one or two branches of the left coronary artery and correction of mitral/tricuspid valve or aortic valve pathologies [10]. However, the number of patients receiving a minimally invasive heart operation is certainly influenced by a strong selection bias, since experience and surgical expertise affect patient selection.

In the coronary field, a left anterolateral mini-thoracotomy in the fifth intercostal space is the standard access point when grafting the LIMA to the LAD [11]. This procedure is generally performed off-pump. Advancements in this technique led to the performance of multiple bypass grafting through the same approach [12], and some specialized centers even use both internal thoracic arteries when using this approach [13,14]. Telemanipulators offer great visibility and maneuverability for ITA harvesting and are favored by some surgeons [15]. Although MIDCAB approaches allow all coronary vessels to be targeted, the LAD is the best target and these techniques are often used in the context of additional coronary stenting (also known as hybrid procedures) [16]. Most recently, anterior throacotomies (TCRAT) have been proposed [17] to perform complete bypass operations including the use of cardiopulmonary bypass and classic aortic clamp techniques, allowing secure grafting of all coronary targets without the need to operate off-pump.

Minimally invasive approaches require cardiopulmonary bypass (except for off-pump CABG cases) which is often established through peripheral (mostly groin) cannulation. Despite the initial skepticism regarding a presumed increase in stroke rates [18] (for return of flow in the descending artery) this technique has proven its safety [19]. Percutaneous cannulation of the femoral vessels, among other benefits, accelerates the operation and reduces the number of peri-operative complications [20]. Surgical techniques and myocardial protection generally align with those used in conventional, sternotomy cardiac surgery, with some technical differences, such as the use of specialized long-shafted instruments and retractors. It is important to realize that it has always been the goal, with any of the minimal-access approaches to the heart, to deliver the same surgical procedure to the heart, just through a smaller incision. Advantages may come from causing less surgical trauma and disadvantages may come from the greater surgical challenge of performing the same procedure through a smaller incision and from potentially longer operating times. Thus, assessing the impact of using minimal-access approaches to the heart on outcomes is important.

## 3. Scientific Evidence

Minimally invasive cardiac surgery, when compared to traditional approaches, exhibits fundamentally different characteristics, with these differences being more or less noticeable at all perioperative stages, from the actual surgery itself to the early postoperative period and intermediate outcomes. In general, randomized evidence is scarce. The available randomized trials are summarized in Table 1. In minimally invasive surgery, operative, cardiopulmonary bypass, and aortic cross-clamp times have been described to be longer [21,22,23]. We and others demonstrated that longer aortic cross-clamp times increase the risk of perioperative mortality in conventional sternotomy cardiac surgery [24,25]. However, despite longer myocardial ischemia times for minimally invasive procedures, no difference in mortality was observed compared to the same procedures performed through sternotomy in several investigations [26]. To the contrary, despite longer operative times, early operative secondary endpoints such as ventilation times [22] or right ventricular function [27] were better in the minimally invasive cardiac surgery group. These differences further translated into a trend towards shorter patient stay in the intensive care unit and earlier discharge from the hospital [22]. We recently demonstrated that the relationship of cross-clamp time and mortality can also be found in minimally invasive mitral valve surgery [24], suggesting that this mini-approach must convey some protective effects to counteract the negative impact of elongated clamp times and explain the above-described advantages.

Randomized trials have yielded contradictory results regarding patients’ quality of life and satisfaction at different time points after surgery. The most recent Mini-Mitral trial reported better quality of life and satisfaction at 1 month for patients undergoing minimally invasive mitral valve surgery versus sternotomy, although the primary endpoint at three months was no different [28]. Other reports support the absence of a difference at 3 months [23]. A possible explanation for this observation might be that the influence of the surgical approach becomes less important as time after surgery increases. In the initial postoperative course, sternotomy-free approaches may benefit from quicker physical recovery compared to sternotomy, where bone and wound healing usually takes longer. Postoperative healing is the primary focus in the initial phase, while freedom from symptoms and durability of the cardiac procedure counts in the long-run. Unfortunately, long-term comparisons or functional analyses of thoracic integrity between sternotomy and mini-thoracotomy approaches are not available. However, the above-described goal of delivering the same surgical results through minimized access makes these findings only plausible. A long-term survival benefit for patients undergoing minimally invasive cardiac surgery may not be expected if the surgical result is intended to be the same.

The interventional field is the area in which the “non-inferiority concept” has been the basis of their development. Classic surgical thinking has been guided by the logic that change is required if results are superior. With the development of interventional techniques, change was massive in all areas with an approach for which not being worse was the primary target. The current situation is therefore characterized by steadily growing numbers of interventional techniques and shrinking numbers in classic cardiac surgery in both coronary and valve procedures [7]. The question of whether these transcatheter interventions hold their promise of not being worse has sparked a great discussion in the community [32].

**Figure 3:**
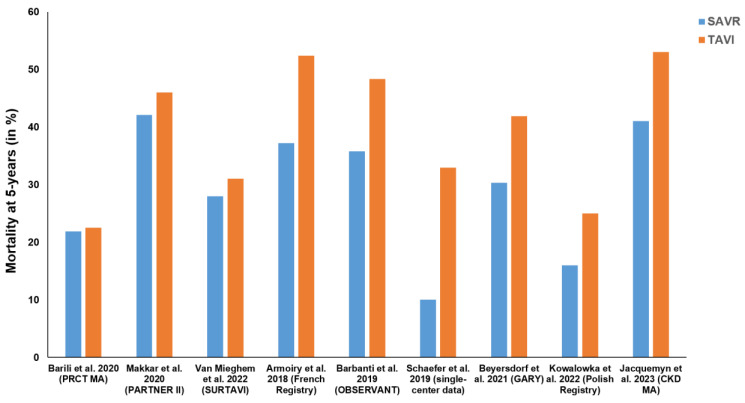
Composition of mortality rates of SAVR and TAVI comparisons from randomized studies and propensity matched registry analyses demonstrating 5-year outcomes. (SAVR—surgical aortic valve replacement, TAVI—transcatheter aortic valve implantation) [35,40,41,42,43,44,45,46,47].

**Figure 4:**
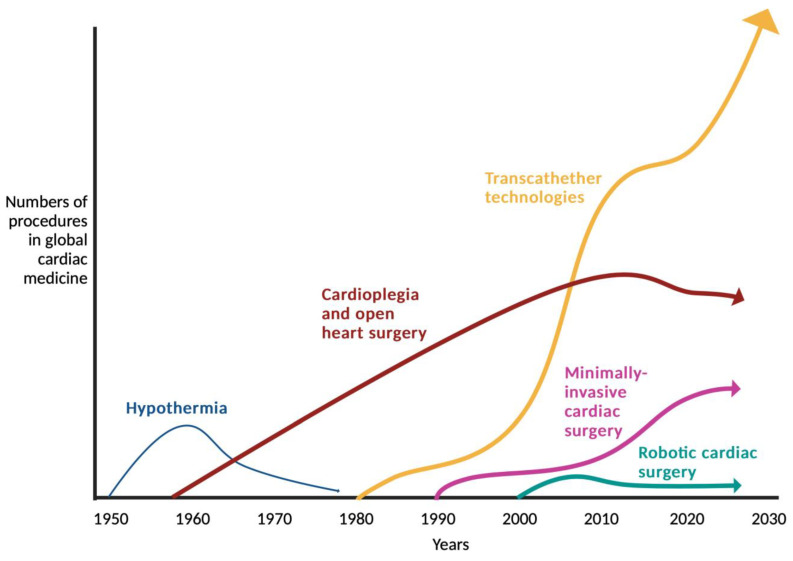
Simplified illustration of the development of the invasive treatment of heart disease with surgical and interventional means over the last 70 years.

Interestingly, the ability of an interventional approach to allow a procedure to be performed without general anesthesia and cardiopulmonary bypass and via a purely percutaneous access route even raised the expectations of improved results, although the statistical designs have always tested for non-inferiority [33]. A prime example to support this statement is the US Corevalve trial that indeed demonstrated the superiority of transcatheter Corevalve placement over classic surgical aortic valve replacement (SAVR) over a three-year period [34]. However, none of the other TAVI vs. SAVR trials could statistically repeat such superiority [35,36]. Any differences that may have been initially observed in favor of TAVI disappeared (mainly in the first year), the latest after 5 years, and a significant survival advantage of SAVR emerged in both randomized and non-randomized evidence for SAVR in the long-run (Figure 3). Since most trials do not follow their patients long-term, the available information sparks the concern that techniques with potentially inferior long-term outcomes are favored over their superior alternatives for the perceived lesser invasiveness. A similar development can be seen in other areas where interventional techniques are increasingly being used [37]. Often times, new interventions are even being tested against medial therapy, although surgery is considered the current gold standard [38,39]. Since most patients are more afraid of classic sternotomy operations today than they may have been before the availability of interventional alternatives, sternotomy-free approaches move into the center of attention in classic cardiac surgery. The development of these approaches, specifically at a time at which valve interventions emerge, underscores this statement. Figure 4 illustrates the development of cardiac surgery over the past 70 years together with the advent of interventional and minimally invasive surgical procedures. Thus, for optimal patient care, heart team discussions are required to provide recommendations that balance short-term outcomes and long-term results.

## 4. Minimally Invasive Cardiac Surgery Requires Specific Training

The increasing demand for minimally invasive cardiac surgical procedures requires surgeons to adapt not only to the different approach, but also to the different tools needed to treat these cases (e.g., specific instruments, techniques, and imaging modalities), different learning curves and different ways to teach these techniques to the young.

Compared to conventional sternotomy, minimally invasive approaches are technically more demanding. There is less room for error, because minor complications may require a conversion to sternotomy or aggravate the course of the operation significantly. The limited visibility through the small incision also limits a teacher’s ability to supervise and correct the performance of surgical trainees. Here, endoscopic and robotically assisted approaches provide a substantial advantage. Specifically, the master–slave concept with the da Vinci system requires mentioning; in this approach, a junior and a senior surgeon sit on two separate consoles and the senior can (similar to a driving instructor) supervise, guide, and even take over the surgical procedure performed by the junior surgeon. However, if the interest in teaching is high enough, with direct supervision, a team of junior and senior surgeons can perform and teach a case safely [48].

Since a minimally invasive procedure usually requires more time even in experienced hands, simulator training becomes more and more attractive and important in order to minimize learning curves and optimize the speed of performance. In addition, familiarizing novices with repair concepts in theory further adds to the safety of surgery by increasing the likelihood that initial repairs will immediately generate success (that is why we have created the motto “cardiac surgery is thinking with your hands” [1]).

The need to reorganize training and qualification for minimally invasive cardiac surgery finds evidence in the literature by studies demonstrating that outcomes of mitral valve surgery are related to the specific expertise of surgeons and centers [49], and that learning curves are long and may be surgeon specific [50]. It may therefore be necessary to rethink our current approach of training novice surgeons through sternotomy approaches first and then expose them to the minimal access later. Individual experiences support the notion that primary training with minimally invasive access is reasonable and safe. In any case, specialized centers (for instance for valve repair surgeries) have the potential to routinely provide outcomes that are excellent [51,52]. In addition, different approaches in general usually expand a field’s horizon. In line with this statement, there is evidence that the minimally invasive approach to cardiac valves also broadened our surgical horizon and provides new perspectives.

## 5. Perspectives of Minimally Invasive Cardiac Surgery

In addition to maintaining thoracic cage integrity, faster recovery and better quality of life in the early recovery period, experience with minimally invasive approaches may allow us to perform procedures that have been considered too risky through (often times redo-) sternotomy. A prime example is the presence of mitral valve regurgitation or stenosis in patients who have previously undergone aortic valve surgery or coronary artery bypass grafting. If the aortic valve is competent, the procedure may be performed via thoracotomy on the beating and/or fibrillating heart, without the need to dissect fragile bypass grafts or divide severe adhesions from previous sternotomies [53,54] (given the required surgical experience is present [21]). Considering that such approaches may allow the establishment of a mechanical result that has the currently best potential for long-term durability, these options are valuable, since the currently available interventional alternatives appear to have not demonstrated their long-term potential and the initial results are questionable [55].

Table 2 summarizes the scenarios in which minimally invasive approaches have the potential to provide advantages for the conduct of classic cardiac surgery. With today’s patients presenting with increased age and number of comorbidities, it must be the goal for surgical procedures to correct the required problem without causing additional harm. Since an operation on a patient without additional morbidities and normal peripheral organ function will likely be the same as addressing the same pathology in a patient with substantial comorbidities and peripheral organ dysfunction, the additional risk in the latter patient is likely to be caused by the trauma created by anesthesia and surgery alike. It appears less dependent on the specific surgical procedure. Thus, in today’s cardiac surgery, the outcomes are not only related to surgical expertise but also to the presence of excellent competencies in areas such as anesthesia, ICU care, perfusion, and physical rehabilitation. In other words, cardiac surgery today is a team effort. Interventional techniques are beginning to replace surgical procedures to some degree, but they are also a valuable adjunct to the current practice of cardiac surgery. Heart team recommendations are becoming the standard for individualized decision making. In these discussions, minimally invasive access surgery has gained a growing role, in which recommendations not only consist of an interventional or surgical approach, but they may also consist of hybrid approaches, minimally invasive versus sternotomy approaches, or even staged procedures. Thus, the options to balance peri-procedural risk and long-term benefits have never been greater than they are today, but it is up to us to master the surgical and interventional skills and work together in order to deliver those skills to the individual patient.

## Figures and Tables

**Table 1:** Summary of current randomized data comparing conventional, less invasive, or interventional procedures for heart valve diseases.

Author	Journal/ Year	Valve	Comparison	Number of Randomized Observations	Result	Mortality
Rodríguez-Caulo et al. [28]	STCVS 2021	Aortic	Sternotomy vs. MICS	100	Better QOL at 1 year in MIC arm	No difference
Vukovic et al. [29]	JCS 2019	Aortic	Sternotomy vs. MICS	100	Lower hospital stay in MICS arm	No difference
Hancock et al. [30]	BMJ 2021	Aortic	Sternotomy vs. MICS	270	Equal transfusions rate	No difference
Dalen et al. [27]	ICVTS 2018	Aortic	Sternotomy vs. MICS	40	Higher postoperative TAPSE in MICS arm	No difference
Feldman et al. [31]	NEJM 2011	Mitral	Sternotomy vs. MitraClip	279	Less re-do surgeries and residual MR in surgical arm	No difference
Nasso et al. [22]	Cardiology 2014	Mitral	Sternotomy vs. MICS	160	Longer operative, bypass and cross-clamp times, but shorter ventilation, ICU and in-hospital stay in MICS arm	No difference
Akowuah et al. [23]	2023	Mitral	Sternotomy vs. MICS	330	No difference in QOL in 3 months	Lower in MICS

**Table 2:** The potential of minimally invasive approaches to provide advantages for the conduct of classic cardiac surgery.

Surgical Scenarios in Which Minimally Invasive Approaches Have Provided Advantages for the Conduct of Classic Cardiac Surgery through Sternotomy (Modified from Doenst and Lamelas [21])
Tricuspid valve: surgery without sternotomy, as a redo without pericardial dissection, with or without cross-clamping
Mitral valve: surgery without sternotomy, as a redo (specifically with patent mammary) with or without pericardial dissection, with or without cross-clamping, beating heart/fibrillating heart.
Redo cases with previous sternal wound infection (specifically those with loss of sternal bone)
Cases with morbid obesity
Frail patients with or without significant osteoporosis
Patients with large breast implants

## Data Availability

Represented data available in a publicly accessible biomedical databases.

## Abbreviations

CABG	Coronary Artery Bypass Grafting.
CKD	Chronic Kidney Disease.
ICU	Intensive Care Unit.
LAD	Left Anterior Descending.
LIMA	Left Internal Mammary Artery.
MA	Meta-Analysis.
MICS	Minimally Invasive Cardiac Surgery.
MIDCAB	Minimally Invasive Direct Coronary Artery Bypass.
PRCT	Prospective Randomized Clinical Trial.
QOL	Quality of Life.
SAVR	Surgical Aortic Valve Replacement.
TAPSE	Tricuspid Annular Plane Systolic Excursion.
TAVI	Transcatheter Aortic Valve Implantation.
TCRAT	Total coronary revascularization via left anterior thoracotomy.
